# 2-Methyl-5-[(3-methyl-4-nitro­benz­yl)sulfan­yl]-1,3,4-thia­diazole

**DOI:** 10.1107/S1600536810052505

**Published:** 2010-12-24

**Authors:** Hoong-Kun Fun, Suchada Chantrapromma, B. Chandrakantha, Arun M. Isloor, Prakash Shetty

**Affiliations:** a X-ray Crystallography Unit, School of Physics, Universiti Sains Malaysia, 11800 USM, Penang, Malaysia; b Crystal Materials Research Unit, Department of Chemistry, Faculty of Science, Prince of Songkla University, Hat-Yai, Songkhla 90112, Thailand; c Department of Chemistry, Manipal Institute of Technology, Manipal 576 104, India; d Organic Chemistry Division, Department of Chemistry, National Institute of Technology-Karnataka, Surathkal, Mangalore 575 025, India; e Department of Printing, Manipal Institute of Technology, Manipal 576 104, India

## Abstract

The mol­ecule of the title thia­diazole derivative, C_11_H_11_N_3_O_2_S_2_, has a butterfly-like structure and the whole mol­ecule is disordered with a site-occupancy ratio of 0.629 (4):0.371 (4). The mol­ecule is disordered in such a way that the 3-methyl-4-nitro­phenyl units of the major and minor components are approximately related by 180° rotation around the C—N bond axis. The dihedral angle between the 1,3,4-thia­diazole and benzene rings is 70.8 (4)° in the major component and 74.9 (6)° in the minor component. In the crystal, mol­ecules are arranged into screw chains along the *c* axis. These chains are stacked along the *b* axis. Weak inter­molecular C—H⋯O and C—H⋯π inter­actions and a short C⋯O contact [3.005 (7) Å] are present.

## Related literature

For bond-length data, see: Allen *et al.* (1987 ▶). For related structures, see: Fun *et al.* (2011 ▶); Wang *et al.* (2010 ▶). For background to and applications of thia­diazole derivatives, see: Bernard *et al.* (1985 ▶); Chandrakantha *et al.* (2010 ▶); Isloor *et al.* (2010 ▶); Kalluraya *et al.* (2004 ▶); Oruç *et al.* (2004 ▶); Salimon *et al.* (2010 ▶). For the stability of the temperature controller used in the data collection, see: Cosier & Glazer (1986 ▶).
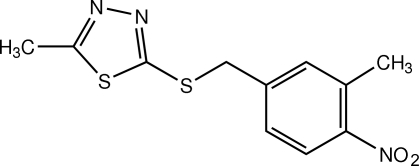


## Experimental

### 

#### Crystal data

C_11_H_11_N_3_O_2_S_2_
*M* *_r_* = 281.37
Orthorhombic, 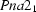

*a* = 13.8210 (14) Å
*b* = 4.5720 (5) Å
*c* = 19.7929 (19) Å
*V* = 1250.7 (2) Å^3^
*Z* = 4
Mo *K*α radiation
μ = 0.42 mm^−1^
*T* = 100 K
0.46 × 0.30 × 0.10 mm

#### Data collection

Bruker APEX DUO CCD area-detector diffractometer
Absorption correction: multi-scan (*SADABS*; Bruker, 2009 ▶) *T*_min_ = 0.831, *T*_max_ = 0.959
10019 measured reflections
3754 independent reflections
3250 reflections with *I* > 2σ(*I*)
*R*_int_ = 0.026

#### Refinement

*R*[*F* ^2^ > 2σ(*F* ^2^)] = 0.031
*wR*(*F* ^2^) = 0.091
*S* = 1.10
3754 reflections
330 parameters
1 restraint
H-atom parameters constrained
Δρ_max_ = 0.45 e Å^−3^
Δρ_min_ = −0.42 e Å^−3^
Absolute structure: Flack (1983 ▶), 1234 Friedel pairs
Flack parameter: 0.04 (6)

### 

Data collection: *APEX2* (Bruker, 2009 ▶); cell refinement: *SAINT* (Bruker, 2009 ▶); data reduction: *SAINT*; program(s) used to solve structure: *SHELXTL* (Sheldrick, 2008 ▶); program(s) used to refine structure: *SHELXTL*; molecular graphics: *SHELXTL*; software used to prepare material for publication: *SHELXTL* and *PLATON* (Spek, 2009 ▶).

## Supplementary Material

Crystal structure: contains datablocks global, I. DOI: 10.1107/S1600536810052505/is2643sup1.cif

Structure factors: contains datablocks I. DOI: 10.1107/S1600536810052505/is2643Isup2.hkl

Additional supplementary materials:  crystallographic information; 3D view; checkCIF report

## Figures and Tables

**Table 1:** Hydrogen-bond geometry (Å, °) *Cg*1, *Cg*2 and *Cg*3 are the centroids of the S2/C9/N1/N2/C8, C1–C6 and C1*A*–C6*A* rings, respectively

*D*—H⋯*A*	*D*—H	H⋯*A*	*D*⋯*A*	*D*—H⋯*A*
C10—H10*B*⋯O2^i^	0.96	2.58	3.534 (11)	171
C7—H7*B*⋯*Cg*2^ii^	0.97	2.65	3.417 (6)	134
C7—H7*B*⋯*Cg*3^ii^	0.97	2.65	3.489 (6)	145
C7*A*—H7*D*⋯*Cg*2^ii^	0.97	2.63	3.269 (10)	124
C7*A*—H7*D*⋯*Cg*3^ii^	0.97	2.50	3.258 (10)	135
C10*A*—H10*F*⋯*Cg*1^iii^	0.96	2.98	3.683 (18)	132

Symmetry codes: (i) 
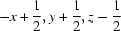
; (ii) 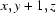; (iii) 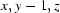.

**Fig. 1.**
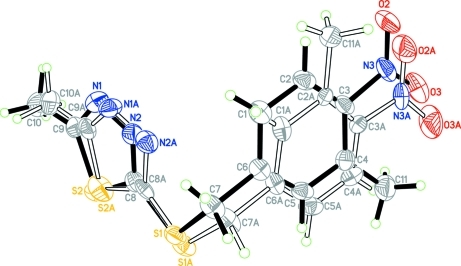
The molecular structure of the title compound, showing 50% probability displacement ellipsoids and the atom-numbering scheme. Open bond show the minor A component.

**Fig. 2.**
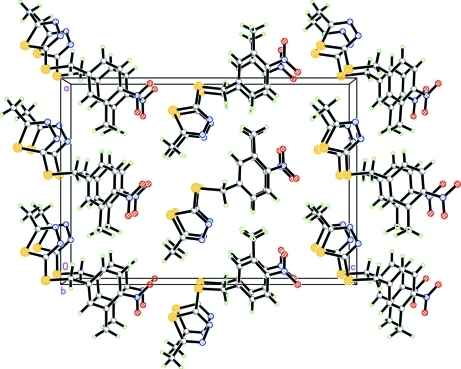
The crystal packing of the title compound viewd along the b axis, showing screw chains running along the c axis.

## supplementary crystallographic information

### Comment

Many classes of thiadiazole compounds have been intensely investigated with a number of them having found to be biologically and pharmacologically active. The 1,3,4-thiadiazole derivatives exhibit a wide spectrum of pharmacological and biological properties such as antituberculosis, anti-inflammatory, antifungal and antibacterial activities (Bernard *et al.*, 1985; Chandrakantha *et al.*, 2010; Isloor *et al.*, 2010; Kalluraya *et al.*, 2004; Oruç *et al.*, 2004; Salimon *et al.*, 2010). The title 1,3,4-thiadiazole derivative, (I), was synthesized in order to study its biological activity. Herein we report the crystal structure of (I).

The whole molecule of (I), C_11_H_11_N_3_O_2_S_2_, is disordered over two sites with the major component and minor *A* components having refined site-occupancy ratio of 0.629 (3)/0.371 (3) and has a butterfly-like structure with a torsion angle C8–S1–C7–C6 = -79.8 (5)° in major component [-79.6 (9)° in minor *A* component]. The molecule is disordered in such a way that the 3-methyl-4-nitrophenyl unit in the major and minor components is related by 180° rotation. The dihedral angle between the 1,3,4-thiadiazole and benzene rings is 70.8 (4)° in the major component [74.9 (6)° in the minor *A* component]. In both components the nitro group is slightly twisted with respect to the attached benzene ring with the torsion angles O2–N3–C3–C2 = 8.4 (4)° and O3–N3–C3–C2 = -172.5 (3)° in the major component [the corresponding values are -12.1 (7) and 168.7 (5)° in the minor *A* component]. The bond distances are of normal values (Allen *et al.*, 1987) and are comparable with the related structures (Fun *et al.*, 2011; Wang *et al.*, 2010).

In the crystal packing (Fig. 2), the molecules are arranged into screw chains along the *c* axis. These chains are stacked along the *b* axis. The crystal is stabilized by C—H···O and C—H···π weak interactions (Table 1). A short C···O contact [3.005 (7) Å; symmetry code: 1/2 + *x*, -1/2 - *y*, *z*] is observed.

### Experimental

The title compound was synthesized by adding 3-methyl-4-nitrobenzylbromide (3.47 g, 0.0151 mol) dropwise to a stirred solution of 5-methyl-1,3,4-thiadiazole-2-thiol (2.00 g, 0.0151 mol) and anhydrous potassium carbonate (4.16 g, 0.03 mol) in dry acetonitrile (50 ml) at room temperature and the reaction mixture was stirred at room temperature for 5 h. After the completion of reaction, the reaction mixture was filtered and the filtrate was concentrated. The crude product was recrystallized with hot ethanol to afford the title compound as yellow solid (2.20 g, 52% yield). Yellow plate-shaped single crystals of the title compound suitable for *x*-ray structure determination were recrystalized from ethanol by the slow evaporation of the solvent at room temperature after several days (M.p. 443–445 K).

### Refinement

All H atoms were positioned geometrically and allowed to ride on their parent atoms, with *d*(C—H) = 0.93 Å for aromatic, 0.97 Å for CH_2_ and 0.96 Å for CH_3_ atoms. The *U*_iso_(H) values were constrained to be 1.5*U*_eq_ of the carrier atom for methyl H atoms and 1.2*U*_eq_ for the remaining H atoms. A rotating group model was used for the methyl groups. The highest residual electron density peak is located at 0.70 Å from C7 and the deepest hole is located at 1.22 Å from S1A. The whole molecule is disordered over two sites with occupancies 0.629 (3) and 0.371 (3). Initially rigidity and similarity restraints were applied. After a steady state was reached, all these restraints were removed before the final refinement.

### Crystal data

**Table:**

C_11_H_11_N_3_O_2_S_2_	*D*_x_ = 1.494 Mg m^−^^3^
*M**_r_* = 281.37	Melting point = 443–445 K
Orthorhombic, *P**n**a*2_1_	Mo *K*α radiation, λ = 0.71073 Å
Hall symbol: P 2c -2n	Cell parameters from 3.12 reflections
*a* = 13.8210 (14) Å	θ = 30.0–2899°
*b* = 4.5720 (5) Å	µ = 0.42 mm^−^^1^
*c* = 19.7929 (19) Å	*T* = 100 K
*V* = 1250.7 (2) Å^3^	Plate, yellow
*Z* = 4	0.46 × 0.30 × 0.10 mm
*F*(000) = 584	

### Data collection

**Table:**

Bruker APEX DUO CCD area-detector diffractometer	3754 independent reflections
Radiation source: sealed tube	3250 reflections with *I* > 2σ(*I*)
graphite	*R*_int_ = 0.026
φ and ω scans	θ_max_ = 33.7°, θ_min_ = 3.1°
Absorption correction: multi-scan (*SADABS*; Bruker, 2009)	*h* = −21→21
*T*_min_ = 0.831, *T*_max_ = 0.959	*k* = −7→6
10019 measured reflections	*l* = −30→21

### Refinement

**Table:**

Refinement on *F*^2^	Secondary atom site location: difference Fourier map
Least-squares matrix: full	Hydrogen site location: inferred from neighbouring sites
*R*[*F*^2^ > 2σ(*F*^2^)] = 0.031	H-atom parameters constrained
*wR*(*F*^2^) = 0.091	*w* = 1/[σ^2^(*F*_o_^2^) + (0.0501*P*)^2^] where *P* = (*F*_o_^2^ + 2*F*_c_^2^)/3
*S* = 1.10	(Δ/σ)_max_ = 0.001
3754 reflections	Δρ_max_ = 0.45 e Å^−^^3^
330 parameters	Δρ_min_ = −0.42 e Å^−^^3^
1 restraint	Absolute structure: Flack (1983), 1234 Friedel pairs
Primary atom site location: structure-invariant direct methods	Flack parameter: 0.04 (6)

### Special details

**Table:**

Experimental. The crystal was placed in the cold stream of an Oxford Cryosystems Cobra open-flow nitrogen cryostat (Cosier & Glazer, 1986) operating at 100.0 (1) K.
Geometry. All e.s.d.'s (except the e.s.d. in the dihedral angle between two l.s. planes) are estimated using the full covariance matrix. The cell e.s.d.'s are taken into account individually in the estimation of e.s.d.'s in distances, angles and torsion angles; correlations between e.s.d.'s in cell parameters are only used when they are defined by crystal symmetry. An approximate (isotropic) treatment of cell e.s.d.'s is used for estimating e.s.d.'s involving l.s. planes.
Refinement. Refinement of *F*^2^ against ALL reflections. The weighted *R*-factor *wR* and goodness of fit *S* are based on *F*^2^, conventional *R*-factors *R* are based on *F*, with *F* set to zero for negative *F*^2^. The threshold expression of *F*^2^ > σ(*F*^2^) is used only for calculating *R*-factors(gt) *etc*. and is not relevant to the choice of reflections for refinement. *R*-factors based on *F*^2^ are statistically about twice as large as those based on *F*, and *R*- factors based on ALL data will be even larger.

### Fractional atomic coordinates and isotropic or equivalent isotropic displacement parameters (Å^2^)

**Table:**

	*x*	*y*	*z*	*U*_iso_*/*U*_eq_	Occ. (<1)
S1	0.03130 (17)	0.3490 (8)	0.96362 (19)	0.0300 (3)	0.629 (4)
S2	0.1578 (2)	−0.0159 (8)	0.8790 (3)	0.0328 (6)	0.629 (4)
O2	−0.0136 (2)	−0.4228 (6)	1.29413 (14)	0.0518 (8)	0.629 (4)
O3	−0.1559 (3)	−0.4789 (7)	1.25002 (19)	0.0478 (9)	0.629 (4)
N1	0.2757 (4)	−0.0884 (15)	0.9791 (3)	0.0420 (11)	0.629 (4)
N2	0.2002 (4)	0.0921 (19)	1.0023 (4)	0.0346 (12)	0.629 (4)
N3	−0.0749 (2)	−0.3679 (6)	1.25069 (13)	0.0349 (6)	0.629 (4)
C1	0.0766 (3)	0.1353 (6)	1.14831 (15)	0.0289 (5)	0.629 (4)
H1A	0.1410	0.1947	1.1471	0.035*	0.629 (4)
C2	0.0461 (3)	−0.0654 (6)	1.19695 (14)	0.0289 (5)	0.629 (4)
H2A	0.0897	−0.1386	1.2285	0.035*	0.629 (4)
C3	−0.0489 (4)	−0.1543 (6)	1.19794 (14)	0.0262 (5)	0.629 (4)
C4	−0.1181 (3)	−0.0516 (6)	1.15221 (16)	0.0278 (5)	0.629 (4)
C5	−0.0843 (3)	0.1537 (7)	1.10453 (16)	0.0266 (6)	0.629 (4)
H5A	−0.1281	0.2301	1.0735	0.032*	0.629 (4)
C6	0.0110 (3)	0.2467 (7)	1.10173 (18)	0.0244 (5)	0.629 (4)
C7	0.0414 (3)	0.4654 (12)	1.0497 (3)	0.0319 (9)	0.629 (4)
H7A	0.1082	0.5185	1.0583	0.038*	0.629 (4)
H7B	0.0026	0.6404	1.0556	0.038*	0.629 (4)
C8	0.1361 (6)	0.136 (2)	0.9548 (6)	0.0258 (13)	0.629 (4)
C9	0.2662 (7)	−0.157 (3)	0.9161 (5)	0.0336 (14)	0.629 (4)
C10	0.3367 (8)	−0.341 (2)	0.8795 (5)	0.050 (2)	0.629 (4)
H10A	0.3740	−0.4522	0.9113	0.075*	0.629 (4)
H10B	0.3791	−0.2182	0.8535	0.075*	0.629 (4)
H10C	0.3029	−0.4718	0.8498	0.075*	0.629 (4)
S1A	0.0080 (3)	0.3638 (14)	0.9625 (4)	0.0331 (7)	0.371 (4)
S2A	0.1561 (5)	−0.0237 (15)	0.8860 (6)	0.0398 (14)	0.371 (4)
O2A	−0.0852 (4)	−0.5184 (9)	1.2745 (3)	0.0507 (12)	0.371 (4)
O3A	−0.2234 (4)	−0.4513 (11)	1.2302 (3)	0.0600 (14)	0.371 (4)
N1A	0.2532 (5)	−0.046 (2)	0.9912 (4)	0.0311 (14)	0.371 (4)
N2A	0.1738 (7)	0.115 (3)	1.0101 (6)	0.0321 (15)	0.371 (4)
N3A	−0.1378 (4)	−0.3973 (13)	1.2341 (3)	0.0327 (11)	0.371 (4)
C1A	0.0286 (4)	0.1374 (9)	1.1504 (2)	0.0254 (8)	0.371 (4)
H1B	0.0910	0.2118	1.1541	0.031*	0.371 (4)
C2A	−0.0029 (4)	−0.0698 (9)	1.1981 (2)	0.0196 (7)	0.371 (4)
C3A	−0.0968 (4)	−0.1718 (9)	1.1884 (2)	0.0232 (8)	0.371 (4)
C4A	−0.1560 (4)	−0.0847 (11)	1.1368 (3)	0.0292 (10)	0.371 (4)
H4B	−0.2178	−0.1628	1.1322	0.035*	0.371 (4)
C5A	−0.1220 (5)	0.1226 (12)	1.0913 (3)	0.0320 (11)	0.371 (4)
H5B	−0.1613	0.1869	1.0562	0.038*	0.371 (4)
C6A	−0.0289 (5)	0.2336 (11)	1.0987 (3)	0.0238 (10)	0.371 (4)
C7A	0.0060 (7)	0.479 (2)	1.0507 (5)	0.0379 (19)	0.371 (4)
H7C	0.0705	0.5389	1.0640	0.045*	0.371 (4)
H7D	−0.0365	0.6463	1.0553	0.045*	0.371 (4)
C8A	0.1157 (10)	0.166 (4)	0.9600 (8)	0.0220 (18)	0.371 (4)
C9A	0.2489 (11)	−0.138 (4)	0.9296 (7)	0.0294 (19)	0.371 (4)
C10A	0.3250 (10)	−0.317 (4)	0.8965 (8)	0.043 (3)	0.371 (4)
H10D	0.3736	−0.3676	0.9291	0.065*	0.371 (4)
H10E	0.3539	−0.2066	0.8606	0.065*	0.371 (4)
H10F	0.2966	−0.4921	0.8786	0.065*	0.371 (4)
C11	−0.2234 (2)	−0.1340 (8)	1.14972 (19)	0.0438 (7)	0.629 (4)
H11A	−0.2292	−0.3416	1.1436	0.066*	0.629 (4)
H11B	−0.2540	−0.0349	1.1127	0.066*	0.629 (4)
H11C	−0.2541	−0.0783	1.1913	0.066*	0.629 (4)
C11A	0.0647 (3)	−0.1528 (12)	1.2541 (2)	0.0357 (11)	0.371 (4)
H11D	0.1235	−0.0425	1.2500	0.053*	0.371 (4)
H11E	0.0790	−0.3579	1.2513	0.053*	0.371 (4)
H11F	0.0348	−0.1111	1.2968	0.053*	0.371 (4)

### Atomic displacement parameters (Å^2^)

**Table:**

	*U*^11^	*U*^22^	*U*^33^	*U*^12^	*U*^13^	*U*^23^
S1	0.0368 (8)	0.0311 (6)	0.0220 (4)	0.0045 (6)	−0.0004 (8)	0.0056 (4)
S2	0.0371 (10)	0.0316 (9)	0.0298 (14)	0.0018 (7)	−0.0078 (6)	0.0015 (6)
O2	0.0612 (16)	0.0623 (16)	0.0319 (13)	0.0179 (13)	0.0060 (11)	0.0219 (12)
O3	0.067 (2)	0.0426 (16)	0.0343 (17)	−0.0141 (14)	0.0107 (15)	0.0050 (11)
N1	0.032 (2)	0.051 (2)	0.043 (3)	0.0138 (15)	0.0031 (15)	0.0084 (18)
N2	0.027 (2)	0.050 (3)	0.027 (2)	−0.003 (2)	−0.0033 (17)	0.0076 (17)
N3	0.0559 (17)	0.0290 (12)	0.0197 (10)	0.0099 (10)	0.0105 (10)	0.0022 (9)
C1	0.0360 (15)	0.0260 (12)	0.0248 (12)	−0.0017 (11)	−0.0003 (11)	−0.0036 (9)
C2	0.0373 (16)	0.0297 (12)	0.0197 (11)	0.0073 (11)	−0.0029 (10)	−0.0019 (9)
C3	0.0370 (17)	0.0252 (12)	0.0163 (11)	0.0075 (13)	0.0031 (11)	0.0012 (9)
C4	0.0294 (14)	0.0300 (12)	0.0241 (13)	0.0047 (11)	0.0033 (11)	−0.0032 (11)
C5	0.0340 (18)	0.0252 (12)	0.0206 (12)	0.0064 (13)	−0.0037 (12)	−0.0014 (10)
C6	0.0324 (14)	0.0178 (10)	0.0230 (11)	−0.0012 (12)	0.0007 (13)	−0.0038 (9)
C7	0.052 (2)	0.0167 (14)	0.0273 (16)	0.0025 (17)	0.005 (2)	−0.0016 (11)
C8	0.030 (3)	0.019 (2)	0.029 (3)	−0.0045 (17)	0.0014 (19)	0.0020 (18)
C9	0.027 (3)	0.0321 (19)	0.041 (4)	−0.0015 (19)	0.0047 (19)	0.011 (2)
C10	0.044 (3)	0.038 (2)	0.068 (6)	0.0098 (17)	0.011 (3)	0.008 (3)
S1A	0.050 (2)	0.0273 (7)	0.0214 (7)	0.0055 (15)	0.0021 (18)	0.0007 (6)
S2A	0.054 (2)	0.042 (2)	0.0236 (17)	−0.0138 (14)	−0.0082 (9)	−0.0056 (12)
O2A	0.063 (3)	0.037 (2)	0.052 (3)	0.0047 (17)	0.016 (2)	0.019 (2)
O3A	0.042 (2)	0.085 (3)	0.053 (3)	−0.029 (2)	0.008 (2)	0.006 (2)
N1A	0.028 (4)	0.038 (3)	0.027 (3)	0.007 (3)	−0.006 (2)	−0.002 (2)
N2A	0.035 (4)	0.036 (3)	0.025 (3)	−0.005 (4)	−0.004 (3)	0.001 (2)
N3A	0.032 (2)	0.035 (3)	0.031 (3)	−0.0079 (18)	0.0141 (19)	−0.0017 (19)
C1A	0.031 (2)	0.0239 (18)	0.0215 (19)	−0.0072 (17)	0.0047 (17)	−0.0059 (14)
C2A	0.0142 (18)	0.0248 (19)	0.0199 (17)	0.0023 (16)	0.0023 (14)	−0.0001 (13)
C3A	0.0221 (19)	0.0241 (18)	0.023 (2)	0.0049 (16)	0.0023 (16)	−0.0002 (15)
C4A	0.022 (2)	0.031 (2)	0.034 (3)	0.0036 (17)	−0.0040 (18)	−0.0013 (18)
C5A	0.039 (3)	0.025 (2)	0.032 (3)	0.010 (2)	−0.005 (2)	0.0006 (18)
C6A	0.034 (3)	0.0163 (17)	0.021 (2)	−0.003 (2)	0.005 (2)	−0.0015 (14)
C7A	0.070 (5)	0.020 (2)	0.024 (2)	0.014 (4)	0.015 (4)	−0.0003 (17)
C8A	0.039 (6)	0.015 (3)	0.012 (2)	−0.004 (3)	0.002 (4)	0.000 (2)
C9A	0.030 (5)	0.028 (4)	0.030 (5)	−0.008 (4)	0.003 (3)	0.010 (3)
C10A	0.030 (4)	0.046 (6)	0.053 (6)	−0.010 (4)	0.000 (4)	0.009 (4)
C11	0.0323 (13)	0.062 (2)	0.0372 (16)	−0.0044 (12)	0.0038 (11)	0.0024 (14)
C11A	0.0303 (18)	0.052 (3)	0.025 (2)	0.0046 (17)	−0.0020 (16)	0.0000 (18)

### Geometric parameters (Å, °)

**Table:**

S1—C8	1.756 (7)	S2A—C8A	1.791 (17)
S1—C7	1.791 (8)	O2A—N3A	1.213 (9)
S2—C8	1.678 (12)	O3A—N3A	1.211 (7)
S2—C9	1.790 (12)	N1A—C9A	1.291 (16)
O2—N3	1.233 (4)	N1A—N2A	1.376 (11)
O3—N3	1.229 (5)	N2A—C8A	1.30 (2)
N1—C9	1.293 (11)	N3A—C3A	1.485 (7)
N1—N2	1.407 (7)	C1A—C6A	1.368 (8)
N2—C8	1.306 (14)	C1A—C2A	1.407 (6)
N3—C3	1.474 (4)	C1A—H1B	0.9300
C1—C6	1.390 (5)	C2A—C3A	1.391 (7)
C1—C2	1.396 (4)	C2A—C11A	1.498 (7)
C1—H1A	0.9300	C3A—C4A	1.369 (7)
C2—C3	1.374 (6)	C4A—C5A	1.389 (8)
C2—H2A	0.9300	C4A—H4B	0.9300
C3—C4	1.397 (5)	C5A—C6A	1.391 (8)
C4—C5	1.410 (4)	C5A—H5B	0.9300
C4—C11	1.504 (5)	C6A—C7A	1.546 (11)
C5—C6	1.385 (4)	C7A—H7C	0.9700
C5—H5A	0.9300	C7A—H7D	0.9700
C6—C7	1.495 (7)	C9A—C10A	1.48 (2)
C7—H7A	0.9700	C10A—H10D	0.9600
C7—H7B	0.9700	C10A—H10E	0.9600
C9—C10	1.476 (13)	C10A—H10F	0.9600
C10—H10A	0.9600	C11—H11A	0.9600
C10—H10B	0.9600	C11—H11B	0.9600
C10—H10C	0.9600	C11—H11C	0.9600
S1A—C8A	1.742 (12)	C11A—H11D	0.9600
S1A—C7A	1.822 (13)	C11A—H11E	0.9600
S2A—C9A	1.63 (2)	C11A—H11F	0.9600
			
C8—S1—C7	101.3 (5)	O2A—N3A—C3A	119.3 (5)
C8—S2—C9	86.1 (5)	C6A—C1A—C2A	122.5 (5)
C9—N1—N2	112.4 (7)	C6A—C1A—H1B	118.7
C8—N2—N1	111.0 (6)	C2A—C1A—H1B	118.7
O3—N3—O2	123.3 (3)	C3A—C2A—C1A	115.0 (4)
O3—N3—C3	119.2 (3)	C3A—C2A—C11A	126.8 (4)
O2—N3—C3	117.5 (3)	C1A—C2A—C11A	118.2 (5)
C6—C1—C2	120.1 (3)	C4A—C3A—C2A	124.3 (4)
C6—C1—H1A	120.0	C4A—C3A—N3A	115.4 (5)
C2—C1—H1A	120.0	C2A—C3A—N3A	120.3 (5)
C3—C2—C1	119.6 (3)	C3A—C4A—C5A	118.7 (5)
C3—C2—H2A	120.2	C3A—C4A—H4B	120.7
C1—C2—H2A	120.2	C5A—C4A—H4B	120.7
C2—C3—C4	123.0 (3)	C4A—C5A—C6A	119.6 (5)
C2—C3—N3	116.0 (3)	C4A—C5A—H5B	120.2
C4—C3—N3	121.0 (4)	C6A—C5A—H5B	120.2
C3—C4—C5	115.5 (3)	C1A—C6A—C5A	119.9 (5)
C3—C4—C11	126.8 (3)	C1A—C6A—C7A	120.8 (7)
C5—C4—C11	117.7 (3)	C5A—C6A—C7A	119.2 (7)
C6—C5—C4	123.1 (3)	C6A—C7A—S1A	112.6 (7)
C6—C5—H5A	118.5	C6A—C7A—H7C	109.1
C4—C5—H5A	118.5	S1A—C7A—H7C	109.1
C5—C6—C1	118.8 (3)	C6A—C7A—H7D	109.1
C5—C6—C7	120.0 (3)	S1A—C7A—H7D	109.1
C1—C6—C7	121.2 (3)	H7C—C7A—H7D	107.8
C6—C7—S1	115.7 (4)	N2A—C8A—S1A	126.9 (11)
C6—C7—H7A	108.3	N2A—C8A—S2A	110.2 (9)
S1—C7—H7A	108.3	S1A—C8A—S2A	122.8 (10)
C6—C7—H7B	108.3	N1A—C9A—C10A	124.2 (16)
S1—C7—H7B	108.3	N1A—C9A—S2A	115.5 (11)
H7A—C7—H7B	107.4	C10A—C9A—S2A	120.1 (12)
N2—C8—S2	117.3 (5)	C9A—C10A—H10D	109.5
N2—C8—S1	124.9 (8)	C9A—C10A—H10E	109.5
S2—C8—S1	117.7 (7)	H10D—C10A—H10E	109.5
N1—C9—C10	123.1 (10)	C9A—C10A—H10F	109.5
N1—C9—S2	113.1 (6)	H10D—C10A—H10F	109.5
C10—C9—S2	123.8 (9)	H10E—C10A—H10F	109.5
C8A—S1A—C7A	101.0 (7)	C2A—C11A—H11D	109.5
C9A—S2A—C8A	88.2 (8)	C2A—C11A—H11E	109.5
C9A—N1A—N2A	113.3 (10)	H11D—C11A—H11E	109.5
C8A—N2A—N1A	112.5 (10)	C2A—C11A—H11F	109.5
O3A—N3A—O2A	122.3 (6)	H11D—C11A—H11F	109.5
O3A—N3A—C3A	118.4 (6)	H11E—C11A—H11F	109.5
			
C9—N1—N2—C8	2.8 (11)	C9A—N1A—N2A—C8A	−6.5 (18)
C6—C1—C2—C3	0.7 (4)	C6A—C1A—C2A—C3A	0.6 (6)
C1—C2—C3—C4	−0.3 (4)	C6A—C1A—C2A—C11A	−178.0 (4)
C1—C2—C3—N3	179.7 (2)	C1A—C2A—C3A—C4A	0.5 (6)
O3—N3—C3—C2	−172.5 (3)	C11A—C2A—C3A—C4A	178.9 (4)
O2—N3—C3—C2	8.4 (4)	C1A—C2A—C3A—N3A	178.3 (4)
O3—N3—C3—C4	7.4 (4)	C11A—C2A—C3A—N3A	−3.3 (7)
O2—N3—C3—C4	−171.6 (3)	O3A—N3A—C3A—C4A	−12.1 (7)
C2—C3—C4—C5	−0.5 (4)	O2A—N3A—C3A—C4A	168.7 (5)
N3—C3—C4—C5	179.6 (2)	O3A—N3A—C3A—C2A	169.9 (5)
C2—C3—C4—C11	−179.5 (3)	O2A—N3A—C3A—C2A	−9.3 (7)
N3—C3—C4—C11	0.6 (4)	C2A—C3A—C4A—C5A	−1.2 (7)
C3—C4—C5—C6	0.9 (4)	N3A—C3A—C4A—C5A	−179.1 (5)
C11—C4—C5—C6	−180.0 (3)	C3A—C4A—C5A—C6A	0.8 (8)
C4—C5—C6—C1	−0.6 (5)	C2A—C1A—C6A—C5A	−1.0 (7)
C4—C5—C6—C7	−179.9 (3)	C2A—C1A—C6A—C7A	175.3 (5)
C2—C1—C6—C5	−0.3 (4)	C4A—C5A—C6A—C1A	0.2 (8)
C2—C1—C6—C7	179.1 (3)	C4A—C5A—C6A—C7A	−176.1 (6)
C5—C6—C7—S1	−63.8 (4)	C1A—C6A—C7A—S1A	121.8 (6)
C1—C6—C7—S1	116.9 (4)	C5A—C6A—C7A—S1A	−61.9 (7)
C8—S1—C7—C6	−79.8 (5)	C8A—S1A—C7A—C6A	−79.6 (9)
N1—N2—C8—S2	−1.9 (12)	N1A—N2A—C8A—S1A	−179.7 (12)
N1—N2—C8—S1	−179.9 (7)	N1A—N2A—C8A—S2A	5.2 (17)
C9—S2—C8—N2	0.4 (9)	C7A—S1A—C8A—N2A	2.0 (18)
C9—S2—C8—S1	178.6 (7)	C7A—S1A—C8A—S2A	176.5 (10)
C7—S1—C8—N2	−0.7 (11)	C9A—S2A—C8A—N2A	−2.4 (14)
C7—S1—C8—S2	−178.7 (6)	C9A—S2A—C8A—S1A	−177.7 (12)
N2—N1—C9—C10	179.0 (9)	N2A—N1A—C9A—C10A	179.9 (13)
N2—N1—C9—S2	−2.5 (10)	N2A—N1A—C9A—S2A	4.6 (16)
C8—S2—C9—N1	1.3 (8)	C8A—S2A—C9A—N1A	−1.3 (13)
C8—S2—C9—C10	179.7 (10)	C8A—S2A—C9A—C10A	−176.8 (14)

### Hydrogen-bond geometry (Å, °)

**Table:**

*Cg*1, *Cg*2 and *Cg*3 are the centroids of the S2/C9/N1/N2/C8, C1–C6 and C1A–C6A rings, respectively

**Table:**

*D*—H···*A*	*D*—H	H···*A*	*D*···*A*	*D*—H···*A*
C10—H10B···O2^i^	0.96	2.58	3.534 (11)	171
C7—H7B···Cg2^ii^	0.97	2.65	3.417 (6)	134
C7—H7B···Cg3^ii^	0.97	2.65	3.489 (6)	145
C7A—H7D···Cg2^ii^	0.97	2.63	3.269 (10)	124
C7A—H7D···Cg3^ii^	0.97	2.50	3.258 (10)	135
C10A—H10F···Cg1^iii^	0.96	2.98	3.683 (18)	132

Symmetry codes: (i) −*x*+1/2, *y*+1/2, *z*−1/2; (ii) *x*, *y*+1, *z*; (iii) *x*, *y*−1, *z*.
